# Periodontitis and *Porphyromonas gingivalis* in Preclinical Stage of Arthritis Patients

**DOI:** 10.1371/journal.pone.0122121

**Published:** 2015-04-07

**Authors:** Motomu Hashimoto, Toru Yamazaki, Masahide Hamaguchi, Takeshi Morimoto, Masashi Yamori, Keita Asai, Yu Isobe, Moritoshi Furu, Hiromu Ito, Takao Fujii, Chikashi Terao, Masato Mori, Takashi Matsuo, Hiroyuki Yoshitomi, Keiichi Yamamoto, Wataru Yamamoto, Kazuhisa Bessho, Tsuneyo Mimori

**Affiliations:** 1 Department of the Control for Rheumatic Diseases, Graduate School of Medicine, Kyoto University, Kyoto, Japan; 2 Department of Oral and Maxillofacial Surgery, Graduate School of Medicine, Kyoto University, Kyoto, Japan; 3 Department of Endocrinology and Metabolism, Graduate School of Medical Science, Kyoto Prefectural University of Medicine, Kyoto, Japan; 4 Division of General Medicine, Department of Internal Medicine, Hyogo College of Medicine, Hyogo, Japan; 5 Department of Orthopedic Surgery, Graduate School of Medicine, Kyoto University, Kyoto, Japan; 6 Department of Rheumatology and Clinical Immunology, Graduate School of Medicine, Kyoto University, Kyoto, Japan; 7 Center for Genomic Medicine, Graduate School of Medicine, Kyoto University, Kyoto, Japan; 8 Center for Innovation in Immunoregulative Technology and Therapeutics, Graduate School of Medicine, Kyoto University, Kyoto, Japan; 9 Department of Clinical Epidemiology and Biostatistics, Graduate School of Medicine, Osaka University, Osaka, Japan; 10 Department of Health Information Management, Kurashiki Sweet Hospital, Kurashiki, Japan; Faculté de médecine de Nantes, FRANCE

## Abstract

**Purpose:**

To determine whether the presence of periodontitis (PD) and *Porphyromonas gingivalis* (Pg) in the subgingival biofilm associates with the development of rheumatoid arthritis (RA) in treatment naïve preclinical stage of arthritis patients.

**Methods:**

We conducted a prospective cohort study of 72 consecutive patients with arthralgia who had never been treated with any anti-rheumatic drugs or glucocorticoids. Periodontal status at baseline was assessed by dentists. PD was defined stringently by the maximal probing depth≧4 mm, or by the classification by the 5th European Workshop in Periodontology (EWP) in 2005 using attachment loss. Up to eight plaque samples were obtained from each patient and the presence of Pg was determined by Taqman PCR. The patients were followed up for 2 years and introduction rate of methotrexate (MTX) treatment on the diagnosis of RA was compared in patients with or without PD or Pg.

**Results:**

Patients with PD (probing depth≧4mm) had higher arthritis activity (p = 0.02) and higher risk for future introduction of MTX treatment on the diagnosis of RA during the follow up than patients without PD (Hazard ratio 2.68, p = 0.03). Arthritis activity and risk for MTX introduction increased with the severity of PD assessed by EWP, although not statistically significant. On the other hand, presence of *Pg* was not associated with arthritis activity (p = 0.72) or the risk for MTX introduction (p = 0.45).

**Conclusion:**

In treatment naïve arthralgia patients, PD, but not the presence of Pg, associates with arthritis activity and future requirement of MTX treatment on the diagnosis of RA.

## Introduction

Recent studies have suggested a correlation between periodontitis (PD) and rheumatoid arthritis (RA)[1]. Epidemiological studies have reported the increased prevalence of PD in RA patients[2] [3]. PD and RA share some underlying pathological processes such as involvement of inflammatory cytokines and bone resorption[1]. In addition, recent studies suggested the specific role of a periodontal pathogen, *Porphyromonas gingvalis* (Pg) for anti-citrullinated protein/peptide antibody (ACPA) production for RA [4] [5]. To date, Pg is the only known eubacteria expressing peptidylarginine deaminase that citrullinates human fibrinogen or α-enolase *in vitro* [6] [7] and it is postulated that Pg infection might be a cause of ACPA production and subsequent RA development [4] [5] [8]. However, this hypothesis has not been fully determined *in vivo* yet.

Recent evidences suggested the importance of microbiome in the mucosal surface for the development of autoimmune diseases[9]. For example, a single commensal bacteria, segmented filamentous bacteria, was responsible for the development of inflammatory helper T cell subset (Th17 cells) and for the development of autoimmune arthritis in mice[10]. Presence of clostridia strains in the colon induces regulatory T cells in humans which is important for the maintenance of immune tolerance[11]. Therefore, it is possible that an alteration in the microbiome in the gut or gum may tilt the balance of immune homeostasis and influence the autoimmune disease manifestation. Based on the above mentioned close epidemiological and pathological relationship between PD and RA[1], it is interesting to determine whether the presence of PD or Pg in subgingival biofilm drives the development of RA.

To study the true temporal relationship between PD or Pg and RA, a prospective study of treatment naïve, preclinical stage of arthritis patients is necessary. Immunosuppressive treatments for RA may alter PD status and periodontal biofilm of the patients. In addition, to determine the role of the periodontal pathogens in subgingival biofilm for the development of RA, presence of periodontal pathogens should be directly determined by a DNA sequence-based method[9]. DNA of subgingival biofilm have been analyzed in various diseases such as artherosclerosis or coronary heart diseases which revealed the association between PD/Pg and systemic diseases[12] [13]. However, only a few studies have analyzed the periodontal biofilm in RA patients[14] [15].

In this study, we conducted a prospective cohort study of treatment-naïve arthralgia patient to confirm whether the presence of clinical PD or Pg in subgingival biofilm associates with arthritis activity and future development of RA.

## Methods

### Study design

We conducted a prospective cohort study at the Center for Rheumatic Diseases in conjunction with the Department of Oral and Maxillofacial Surgery in Kyoto University Hospital. Inclusion criteria were the patients aged at least 18 years old when they first visited the center from May 1^st^ in 2011 to December 31^st^ in 2012, who did not have definite diagnosis for arthralgia before the referral to the hospital, and who have never been treated with any synthetic or biologic disease modifying anti-rheumatic drugs or glucocorticoids. Exclusion criteria were patients diagnosed as osteoarthritis without any signs or symptoms of inflammatory synovitis, those who had less than ten teeth remaining, and those who have been treated with antibiotics within one month before the visit. At the first visit to the Center for Rheumatic Diseases, all the patients were referred to the Department of Oral and Maxillofacial Surgery and received full examination of the oral status by trained dentists. The diagnosis of PD was determined by dentists and subgingival plaque samples were obtained and the amount of Pg DNA in biofilm was determined. Then, the study patients were prospectively followed up by rheumatologists for 2 years and initiation of methotrexate (MTX) treatment on the diagnosis of RA was recorded. Rheumatologists and dentists evaluated each patient independently and were blinded from each other for the patients’ dental or rheumatologic status. The study was designed in accordance with the Helsinki declaration and was approved by the ethics committee of Kyoto University Hospital. Written informed consent was obtained from all the patients.

### Assessment of periodontal status and definition for the diagnosis of periodontitis

The periodontal examination was conducted by trained dentists (TY, KA, YI). Calibration of periodontal probing was performed prior to the study. The inter examiner kappa index was 0.7 to 0.9. Clinical data on PD included the number of present teeth, probing depth, attachment loss, bleeding of probing, and plaque control record. Probing depth was measured for all teeth at six sites per tooth (mesio-buccal, misio-lingal, disto-buccal, disto-lingal, mid-buccal and mid-lingal) by UNC-15 probe (Hu-Friedy)[16]. Bleeding of probing was expressed as the percentage of bleeding sites over the total number of tooth surfaces. Oral hygiene was measured using O’Leary’s plaque control record[17]. Presence of PD was defined stringently by the presence of any sites exhibiting probing depth≧4 mm [16] or based upon the classification by the 5^th^ European Workshop in Periodontology (EWP) in 2005 using attachment loss (Moderate PD; presence of proximal attachment loss of ≧3 mm in ≧2 non-adjacent teeth. Severe PD; presence of proximal attachment loss of ≧5 mm in ≧30% of teeth present) [18]. Lifestyle habits that affect PD status such as smoking and tooth brushing were also recorded[19].

### Definition for the presence of *P*. *gingivalis* in subgingival biofilm

To analyze the presence of Pg in subgingival biofilm independent of PD status, subgingival plaque samples were obtained from the mesio-buccal sites of two most posterior teeth in each quadrant as available, irrespective of their periodontal states[12]. Up to eight (average 7.9) subgingival plaque samples were collected for each patient. Teeth were gently dried with sterile cotton swabs. After removing supragingival plaque by cotton pellets and air-drying, subgingival plaque was collected with two sterile paper points inserted into the bottom of the periodontal pocket or gingival crevice for 20 seconds[20]. Bacterial DNA was extracted by Nucleospin tissue XS kit according to the manufacturer’s instructions. Bacterial DNA was not extracted in one patient. Genomic DNA was amplified by specific Taqman primers and probes designed from the variable regions of 16s rRNA of eubacteria gene[21] and quantified by ABI PRISM 7700 Sequence Detection System (Applied Biosystems, Foster City, CA). Presence of Pg in subgingival biofilm was defined stringently as the detection of Pg genomic DNA in at least one site out of eight plaque samples per patient. The bacterial load of Pg was determined by real time PCR normalized with that of total eubacteria gene (*Universal*) [21], summed up for eight plaque samples, and expressed as arbitrary unit. Primers and probes for *Pg* and *Universal* sequence are shown below.

P. gingivalis

Forward; 5’-tgcaacttgccttacagaggg-3’,

Reverse; 5’-actcgtatcgcccgttattc-3’,

Probe; 5’-agctgtaagataggcatgcgtcccattagcta-3’

Universal

Foward; 5’-TCTACGGGAGGCAGCAGT-3’

Reverse; 5’-ggactaccagggtatctaatcctgtt-3’

Probe; 5’-CGTATTACCGCGGCTGCTGGCAC-3’

### Assessment of arthritis status

Clinical and laboratory data on arthritis was obtained by rheumatologists (MH, MF, HI, TF) and stored in the Kyoto University Rheumatoid Arthritis Management Alliance (KURAMA) database [22]. Clinical data included age, sex, disease duration of joint symptoms, swollen joint count, tender joint count, patient’s global assessment of disease activity, and physician’s global assessment of disease activity. Arthritis activity was assessed by simplified disease activity index (SDAI). Physical disability was assessed by modified health assessment questionnaire (mHAQ). Serological data included CRP, ACPA (anti-CCP2 antibody enzyme-linked immunosorbent assay), and rheumatoid factor (latex agglutination turbidimetry).

### Definition of RA diagnosis

RA diagnosis at the first visit was based upon the American College of Rheumatology / European League Against Rheumatism (ACR/EULAR) classification criteria for RA in 2010[23]. The diagnosis of RA at the end of the follow up was based upon the primary rheumatologists’ diagnosis[24].

### Endpoint

We set the endpoint of our study not by the fulfillment of the 2010 ACR/EULAR criteria but by the introduction of MTX treatment on the diagnosis of RA by rheumatologists. MTX is the anchor drug for the treatment of RA which is used for more than 80% of RA patient as the first DMARDs[25]. Because early MTX treatment in probable RA patients prevents the patients from developing into RA fulfilling the 2010 ACR/EULAR criteria[26], it is recommended to consider the initiation of MTX treatment on the suspected diagnosis of RA if the patient has chronic inflammatory synovitis and other differential diagnosis such as psoriatic arthritis were excluded by professional rheumatologist[27].

### Statistical Analyses

Continuous variables were expressed as mean ± SD and categorical variables were expressed as percentages (numbers). The study patients were divided into two groups based on the presence of PD (maximal probing depth ≧ 4 mm) or the presence of *Pg* in subgingival biofilm, and were divided into 3 groups based upon severity of PD (EWP; none, moderate, and severe). The difference of continuous variables between 2 groups were analyzed by Students’ t-test and those among three groups were analyzed by Kruskal-Wallis test. The difference of categorical variables between the two groups were analyzed by Pearson’s qui-squared test and those among three groups were analyzed by Kruskal-Wallis test. The effect the presence of PD (maximal probing depth ≧4 mm or EWP; none, moderate, and severe) or Pg on arthritis activity (SDAI) was analyzed by multivariate analysis of covariance (ANCOVA) adjusted for major confounding factors associated with periodontitis such as age, sex, smoking status, and tooth brushing habit[19]. The introduction rate of MTX treatment in each groups were analyzed by cumulative hazard method. Association between the alteration of the diagnosis for joint symptoms (RA→RA, non RA→RA, and non RA→non RA) and the presence of PD or Pg were analyzed by Pearson’s qui squared test. P values less than 0.05 was considered statistically significant. SPSS statistical package, version 11.0.1 J (SPSS, Inc., Chicago, IL) was used for all statistical analyses.

## Results

### Patients’ characteristics

During the study period, 87 patients were referred to the Center for Rheumatic Diseases as suspect of RA without prior use of any synthetic or biological disease modifying anti-rheumatic drugs or glucocorticoids. Following the exclusion criteria, 14 osteoarthritis patients without any signs or symptoms of inflammatory synovitis and one patient who had less than ten teeth were excluded. No patients were treated with antibiotics within one month. In the end, 72 patients were defined as the study patients and followed up prospectively for 2 years. The baseline characteristics of the study patients are shown in Table 1. Among the study patients, 68.1% (49) of the patients had at least one sites probing depth≧4mm and diagnosed as having PD (probing depth≧4mm). Based on the EWP classification using attachment loss, 9.7% was classified as non PD, 83.3%(60) as moderate PD, and 6.9%(5) as severe PD. After the determination of Pg genomic DNA by Taqman PCR, 59.2% (42) of the patients were defined as Pg positive. By the examination by rheumatologists, 41.7% (30) of the patients fulfilled 2010 ACR/EULAR criteria for RA at baseline (Table 1).

**Table 1:** Baseline characteristics of the study patients.

	N = 72
Age, years	53.4 ± 14.9
Female, %	90.3% (65)
Body mass index, kg/m2	21.9 ± 3.6
Smoking (current or ever), %	29.2% (21)
Number of tooth brushing /day	2.2 ± 0.7
Number of present teeth	24.8 ± 4.1
Probing depth (maximal), mm	4.1 ± 1.2
Attachment loss (maximal), mm	5.1 ± 1.9
Bleeding of probing, %	6.6 ± 7.7
Plaque control record, %	27.5 ± 17.0
Total bacterial load, arbitrary unit	1180 ± 480
P. gingivalis positive, %	59.2% (42)
P. gingivalis, arbitrary unit	0.2 ± 0.3
PD (probing depth≧4mm)	68.1% (49)
PD (EWP) None	9.7% (7)
PD (EWP) Moderate	83.3% (60)
PD (EWP) Severe	6.9% (5)
Duration of joint symptoms, months	26.8 ± 60.9
Swollen joint count	2.4 ± 3.4
Tender joint count	2.7 ± 3.0
CRP, mg/dl	0.90 ± 1.58
Patient’s global assessment, mm	20.7 ± 23.5
Physician’s global assessment, mm	46.7 ± 28.2
SDAI	12.5 ± 9.8
mHAQ	0.3 ± 0.5
Anti-CCP antibody positive, %	44.9% (31)
Rheumatoid factor positive, %	47.8% (33)
RA diagnosis at baseline, %	41.7% (30)

Continuous variables were expressed as mean ± SD. Categorical variables were expressed as % (number). EWP: periodontitis classification criteria of the 5^th^ European Workshop in Periodontology in 2005, SDAI: simplified disease activity index; mHAQ: modified health assessment questionnaire

### Arthritis activity

At baseline, patients with PD (probing depth≧4mm) had higher arthritis activity than patients without PD (SDAI 14.2±10.4 vs 8.6±7.1, p = 0.02) (Table 2). Arthritis activity such as SDAI, SJC, TJC, and CRP also increased with PD (EWP), although the difference did not reach the statistical cut-off of the statistics (Table 2). Both the diagnosis of PD (probing depth≧4mm) and PD(EWP) well correlated with parameters of PD such as bleeding on probing (p = 0.01 for PD (probing depth≧4mm) and p = 0.002 for PD (EWP)) (Table 2). RA diagnosis by 2010 ACR/EULAR criteria and ACPA/RF status were not different in the study groups.

**Table 2:** Characteristics of the patients divided by the clinical diagnosis of periodontitis or the presence of *Porphyromonas gingivalis* in subgingival biofilm.

	PD (probing depth≧4mm)			PD (EWP)				***P*.*gingivalis***		
	Negative	Positive		None	Moderate	Severe		Negative	Positive	
N	N = 23	N = 49	P	N = 7	N = 60	N = 5	P	N = 29	N = 42	P
Age, years	52.2 ± 17.0	53.9 ± 14.0	0.66	52.3 ± 19.4	53.0 ± 14.4	58.8 ± 10.2	0.73	48.6 ± 15.9	57.0 ± 13.3	0.02*
Female, %	95.7% (22)	87.8% (43)	0.42	100% (7)	93.3% (56)	40% (2)	0.0004*	96.6% (28)	85.7% (36)	0.23
Body mass index, kg/m2	21.2 ± 3.6	22.2 ± 3.5	0.25	21.6 ± 2.7	22.0 ± 3.7	21.6 ± 2.4	0.92	21.7 ± 4.0	22.1 ± 3.3	0.63
Smoking (current or ever), %	21.7% (5)	32.7% (16)	0.41	42.9% (3)	25.0 (%) 15)	60.0% (3)	0.18	31% (9)	28.6% (12)	1
Number of tooth brushing /day	2.5 ± 0.5	2.0 ± 0.7	0.003*	2.6 ± 0.5	2.2 ± 0.7	1.8 ± 0.7	0.81	2.2 ± 0.6	2.1±0.8	0.57
Number of present teeth	25.6 ± 4.0	24.4 ± 4.1	0.27	25.0 ± 4.3	24.8 ± 4.0	23.9 ± 3.9	0.81	25.3 ± 4.5	24.6 ± 3.6	0.51
Probing depth (maximal), mm	2.9 ± 0.5	4.7 ± 1.0	N.A.	2.9 ± 0.3	4.1 ± 1.1	6.0 ± 0.6	0.001*	3.8 ± 1.0	4.3 ± 1.3	0.08
Attachment loss (maximal), mm	4.0 ± 1.2	5.6 ± 2.0	0.0003*	3.4 ± 1.0	5.0 ± 1.5	8.6 ± 2.6	0.0003*	4.7 ± 1.5	5.4 ± 2.1	0.13
Bleeding of probing, %	3.3 ± 5.1	8.2 ± 8.3	0.01*	0.7 ± 0.8	6.4 ± 6.9	16.8 ± 11.2	0.002*	5.3 ± 5.9	7.6 ± 8.8	0.24
Plaque control record, %	24.4 ± 15.9	28.9 ± 17.5	0.3	24.1 ± 15.5	28.0 ± 17.6	26.0 ± 6.6	0.93	31.6 ± 19.9	24.5 ± 14.5	0.08
Total bacterial load, arbitrary unit	1050 ± 470	1240 ± 471	0.04*	980 ± 230	1180 ± 500	1390 ± 400	0.25	1010 ± 450	1290 ± 460	0.005*
P. gingivalis positive, %	52.2% (12)	62.5% (30)	0.45	57.1% (4)	55.9% (33)	100% (5)	0.16	N.D.	N.D.	N.D.
P. gingivalis, arbitrary unit	0.2 ± 0.3	0.3 ± 0.3	0.28	0.2 ± 0.3	0.2 ± 0.3	0.5 ± 0.4	0.09	0.0 ± 0.0	0.4±0.3	N.A.
PD (probing depth≧4mm), %	N.D.	N.D.	N.D.	0% (0)	73.3% (44)	100% (5)	0.0001	62.1% (18)	71.4% (30)	0.45
PD (EWP) None, %	30.4% (7)	0.0% (0)	N.A.	N.D.	N.D.	N.D.	N.D.	10.3% (3)	9.5% (4)	N.A.
PD (EWP) Moderate, %	69.6%(16)	89.8% (44)	N.A.	N.D.	N.D.	N.D.	N.D.	89.7% (26)	78.6% (33)	N.A.
PD (EWP) Severe, %	0.0% (0)	10.2% (5)	N.A.	N.D.	N.D.	N.D.	N.D.	0.0% (0)	11.9% (5)	N.A.
Duration of joint symptoms, months	11.0 ± 17.0	34.3 ± 71.9	0.13	13.1 ± 26.9	30.4 ± 65.0	2.8 ± 1.3	0.16	29.6 ± 69.5	25.5 ± 55.7	0.79
Swollen joint count	1.3 ± 2.2	2.9 ± 3.8	0.07	1.0 ± 1.8	2.4 ± 3.4	4.2 ± 4.3	0.29	1.9 ± 2.5	2.7 ± 3.9	0.31
Tender joint count	2.1 ± 2.3	3.0 ± 3.3	0.27	3.1 ± 3.0	2.4 ± 2.5	5.6 ± 5.4	0.46	2.7 ± 2.3	2.8 ± 3.5	0.86
CRP, mg/dl	0.8 ± 1.5	0.9 ± 1.6	0.81	0.7 ± 1.4	0.9 ± 1.6	1.0 ± 1.1	0.67	0.9 ± 1.8	0.9 ± 1.4	0.86
Patient's global assessment,mm	36.5 ± 27.7	51.3 ± 27.5	0.04*	40.6 ± 28.1	46.1 ± 27.9	62.4 ± 23.3	0.26	43.2 ± 29.1	49 ± 28.1	0.4
Physician's global assessment,mm	11.5 ± 12.5	24.8 ± 26.1	0.03*	12.9 ± 14.8	20.0 ± 22.3	39.4 ± 32.9	0.36	21.3 ± 23.3	20.8 ± 24	0.93
SDAI	8.6 ± 7.1	14.2 ± 10.4	0.02*	9.5 ± 7.8	12.2 ± 9.4	20.0 ± 11.8	0.33	12.0 ± 8.6	12.9 ± 10.7	0.72
mHAQ	0.3 ± 0.5	0.4 ± 0.4	0.44	0.1 ± 0.2	0.4 ± 0.5	0.4 ± 0.4	0.53	0.3 ± 0.5	0.4 ± 0.4	0.41
anti-CCP antibody positive, %	38.1% (8)	47.9% (23)	0.6	50% (3)	43.1% (25)	60.0% (3)	0.74	55.2% (16)	38.5% (15)	0.22
Rheumatoid factor positive, %	47.6% (10)	47.9% (23)	1	50% (3)	46.6% (27)	60.0% (3)	0.84	58.6% (17)	41.0% (16)	0.22
RA diagnosis at baseline, %	43.5% (10)	40.8% (20)	1	42.9% (3)	49.0% (24)	60.0% (3)	0.68	44.8% (13)	40.5% (17)	0.81

Continuous variables were expressed as mean ± SD. The difference of continuous variables between two groups were analyzed by students’ t-test and those among three groups were analyzed by Kruskal-Wallis test. Categorical variables were expressed as percentages (numbers). The difference of categorical variables between two groups was analyzed by Pearson’s qui-squared test and those among three groups were analyzed by Kruskal-Wallis test. EWP: periodontitis classification criteria of the 5th European Workshop in Periodontology in 2005; SDAI: simplified disease activity index; mHAQ: modified health assessment questionnaire; N.D.: not determined; N.A.: not assessed

*: p<0.05 Pg was not analyzed in one patient because DNA of subgingival plaque sample was not successfully extracted.

On the other hand, when the patients were divided into two groups based on the presence or absence of Pg in biofilm, arthritis activity was not different in the two groups (12.9±10.7 vs 12.0±8.6 p = 0.72) (Table 2). RA diagnosis and ACPA/RF status were also not different in the two groups. Age was the only factor that was significantly associated with Pg status (57.0±13.3 vs 48.6±15.9, p = 0.02) (Table 2). Presence of Pg slightly correlated with parameters of PD such as probing depth (p = 0.08), attachment loss (p = 0.13), and oral hygiene status (p = 0.08), although they did not reach the significance cut off of the statistics (Table 2).

The presence of PD (maximal probing depth≧4 mm) remained significantly associated with arthritis activity (SDAI) by multivariate analysis (p = 0.02, effect size 0.08–0.09), even after adjustment for major confounding factors associated with PD status such as age, sex, smoking, tooth brushing habit, and the presence of Pg in biofilm (Table 3). In contrast, the presence of PD defined by EWP was not associated with SDAI by multivariate analysis (Table 4).

**Table 3:** Multivariate analysis of covariance for factors associated simplified disease activity index of rheumatoid arthritis.

For SDAI	Model 1		Mode 2		Model 3	
	P	ES	P	ES	P	ES
Age	0.16	0.03	0.15	0.03	0.16	0.03
Women	0.01*	0.10	0.01*	0.09	0.01*	0.11
Number of tooth brushing /day	0.19	0.03	0.62	0.00	0.21	0.03
Current smoker	0.82	0.00	0.66	0.00	0.87	0.00
PD (probing depth≧4mm)	0.02*	0.08	N.D.	N.D.	0.02*	0.09
P. gingivalis positive	N.D.	N.D.	0.68	0.00	0.65	0.00

Multivariate analysis of covariance was used to analyze the effect size of the clinical diagnosis of periodontitis based upon maximal probing depth≧4 mm and the presence of *P*. *gingivalis* for disease activity of rheumatoid arthritis after adjusting for age, sex, smoking status, frequency of tooth brushing per a day. Disease activity of rheumatoid arthritis was evaluated by simplified disease activity index. SDAI; simplified disease activity index, P; P value, ES; Effect size

*; p<0.05

**Table 4:** Multivariate analysis of covariance for factors associated simplified disease activity index of rheumatoid arthritis.

For SDAI	Model 4		Model 5	
	P	ES	P	ES
Age	0.14	0.03	0.15	0.04
Women	0.04*	0.06	0.05*	0.06
Number of tooth brushing /day	0.52	0.01	0.63	0.00
Current smoker	0.56	0.01	0.49	0.01
PD (probing depth ≧ 4mm)	0.69	0.01	0.65	0.01
P. gingivalis positive	N.D.	N.D.	0.50	0.01

Multivariate analysis of covariance was used to analyze the effect size of the clinical diagnosis of periodontitis based upon the 5th European Workshop in Periodontology (EWP) in 2005 (0: no periodontitis, 1: moderate periodontitis, 2: severe periodontitis) and the presence of *P*. *gingivalis* for disease activity of rheumatoid arthritis after adjusting for age, sex, smoking status, frequency of tooth brushing per a day. Disease activity of rheumatoid arthritis was evaluated by simplified disease activity index. SDAI; simplified disease activity index, P; P value, ES; Effect size

*; p<0.05

### Methotrexate treatment introduction

**Fig 1:**
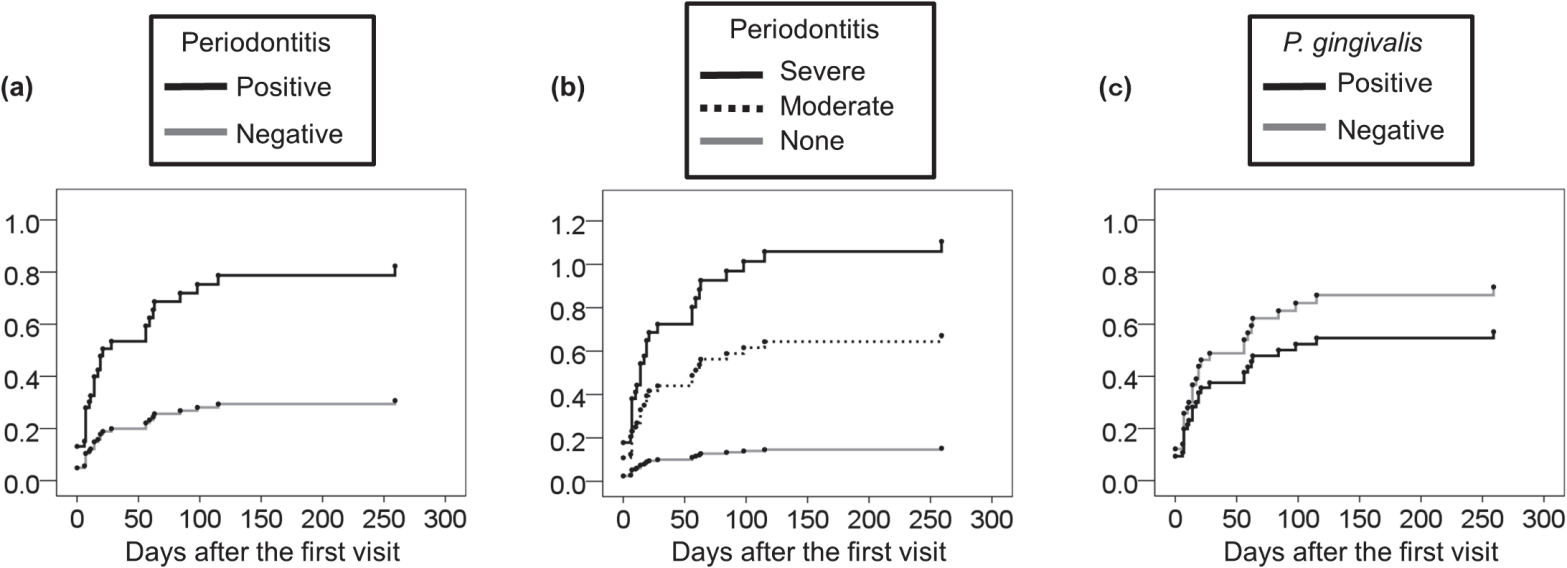
Hazard ratio for methotrexate treatment introduction during the follow up period. The patients were divided into two groups based upon the diagnosis of PD (maximal probing depth ≧ 4 mm) **(a)**, three groups based upon the severity of PD (EWP) **(b)**, or two groups based upon the presence of *P*. *gingivalis* in subgingival biofilm **(c)**. MTX treatment introduction in each groups were compared by cumulative hazard method. X axis indicates days for MTX treatment introduction after the first visit. Y axis indicates the cumulative hazard ratio for MTX introduction. Hazard ratio of positive PD (probing depth≧4mm) vs negative PD was 2.68 (95% CI, 1.11–6.50), p = 0.03. Hazard ratio of severe PD (EWP) vs non PD (EWP) was 7.26 (95% CI, 0.75–69.9), p = 0.09, and hazard ratio of moderate PD vs non PD was 4.42 (95% CI, 0.60–32.4), p = 0.15. Hazard ratio of positive Pg vs negative Pg was 0.77 (95% CI, 0.39–1.53), p = 0.45.

The patients were followed up and the introduction rate of MTX treatment during the follow up period were compared based upon the presence or absence of PD (probing depth≧4mm), severity of PD (EWP), or the presence or absence of Pg at baseline (Fig 1). When the patients were divided into two groups by the presence or absence of PD (probing depth≧4mm), patients with PD (probing depth≧4 mm) were more frequently initiated with MTX treatment than patients without PD (Hazard ratio 2.68 (95% Confidence Interval (CI), 1.11–6.50), p = 0.03) (Fig 1A). When the patients were divided into three groups based on EWP, severe PD (EWP) and moderate PD (EWP) patients had increased risk for the future introduction of MTX treatment compared with non PD patients (Sever vs non PD; Hazard ratio 7.26, (95% CI, 0.75–69.9), p = 0.09, Moderate vs non PD; Hazard ratio 4.42 (95% CI, 0.60–32.4), p = 0.15), although the difference did not reach the significance cut-off of the statistics (Fig 1B).

On the other hand, when the patients were divided into two groups based upon the presence or absence of Pg in subgingival biofilm, hazard ratio for the introduction of MTX treatment was not different in patients with or without Pg in subgingival biofilm (Pg positive vs Pg negative, hazard ratio 0.77 (95% CI, 0.39–1.53), p = 0.45) (Fig 1C).

Relationship between MTX treatment introduction and the alteration of RA diagnosis from baseline to endpoint (RA→RA, non RA→RA, and non RA→non RA) were studied (Table 5). Thirty patients were initially diagnosed as RA by 2010 ACR/EULAR criteria at baseline and also diagnosed as RA by rheumatologists at endpoint (RA→RA). Thirteen patients who did not fulfill 2010 ACR/EULAR criteria at baseline were additionally diagnosed as RA by rheumatologists during the follow up period (non-RA→RA). The remaining 29 patients were classified as non-RA throughout the period (non-RA→non-RA), which included Sjögren syndrome (3), pseudogout (2), polymyalgia rheumatica (1), tenosynovitis (1), and 30.6% (22) patients remained undifferentiated. MTX treatment was introduced in 80.0% of the RA→RA and 76.9% of the non-RA→RA patients but none in the non-RA→non-RA patients. In this study, no patients were initiated with MTX treatment due to other differential diagnosis than RA, such as psoriatic arthritis. Notably, both the presence of PD (probing depth≧4mm) and Pg was more enriched in the disease progression group (non-RA→RA) compared with non-RA (non-RA→non RA) patients (92.3% vs 55.2%, p = 0.04 for PD (probing depth≧4mm) and 61.5% vs 40.0%, p = 0.04 for Pg) (Table 5).

**Table 5:** Diagnosis for joint symptoms at baseline and endpoint.

Baseline→Endpoint	MTX	PD at baseline	Pg at baseline
RA→RA (30)	80.0% (24)	70.0% (21)	43.3% (13)
non RA→RA (13)	76.9% (10)	92.3% (12)	61.5% (8)
non RA→non RA (29)	0.0% (0)	55.2% (16)	40.0% (8)

Diagnosis at baseline is based upon the American College of Rheumatology / European League against Rheumatism (ACR/EULAR) classification criteria for RA in 2010. Diagnosis of RA at endpoint is based upon primary rheumatologists’ diagnosis. Diagnosis at endpoint is based upon the primary rheumatologists’ diagnosis. The diagnosises in non RA→non RA group was Sjogren syndrome (3), Pseudogout (2), Polymyalgia rheumatic (1), Tenosynovitis (1), and Undifferentiated arthritis (22). MTX; methotrexate treatment introduction, PD; diagnosis of periodontitis (maximal probing depth≧4mm), Pg; presence of *Porphyromolas gingivalis* in subgingival biofilm. Categorical variables were expressed as percentages (numbers) and were analyzed by Pearson’s qui squared test. P values for the different PD status groups at baseline are 0.36 (RA→RA vs non RA→non RA), 0.23 (RA→RA vs non RA→RA), 0.04 (non RA→RA vs non RA→non RA), respectively. P values for different Pg status groups at baseline are 0.37 (RA→RA vs non RA→non RA), 0.44 (RA→RA vs non RA→RA), 0.04 (non RA→RA vs non RA→non RA), respectively.

## Discussion

In this study, we showed that in patients with arthralgia who have never been treated with any anti-rheumatic drugs or glucocorticoids, patients with PD had higher arthritis activity and increased risk for future introduction of MTX treatment on the diagnosis of RA than patients without PD. Because this study was performed prospectively on a unique cohort of treatment naïve arthralgia patients, our results suggest that the association between PD and RA is not a mere coincidence, but may have a causal relationship. This study complements and extends the previous studies corroborating the association between PD and RA[1] [14] [28].

In our study, presence of PD (probing depth≧4mm) was associated with arthritis severity and future introduction of MTX treatment. In addition, the severity of PD assessed by EWP classification using attachment loss also correlated with arthritis activity and introduction of MTX treatment, although the difference did not reach the significance cut off the statistics. PD was more enriched in preclinical stage of arthritis (non RA→RA) patients who did not meet the 2010 ACR/EULAR criteria at baseline but later diagnosed as RA by rheumatologists. These results suggest that PD may promote RA development by increasing the joint inflammation. Because DNA of periodontal pathogens are often detected in synovial tissue of RA or atherosclerotic plaque of coronary heart diseases [29] [13], it is possible that periodontal pathogens released from periodontal sites may circulate in the body, reach the synovial tissue, and promote joint inflammation. Activation of innate immunity by periodontal pathogen-derived products provokes chronic joint inflammation in predisposed individuals as is shown by animal models of RA [30] [31].

In contrast with positive correlation between PD and arthritis activity, Pg in subgingival biofilm was not associated with arthritis activity or introduction of MTX treatment. This may be due to several reasons. First, Pg is not the only bacterium that causes PD. PD is a polymicrobial infection caused by a number of pathogens including *Pg*, *Treponema denticola*, and *Tannnerella forsythia* [32] [20]. It is possible that not a single bacterium, but the combination of several periodontal bacteria may be more closely correlated with PD and arthritis activity. It is also possible that not Pg as a whole but some specific genotype of Pg might have a particular virulent factor [33] and might be associated with RA development. Second, not the mere presence of Pg in biofilm but the presence of inflammation in the context of PD may be important for promoting RA development. Indeed, PD and Pg were enriched in the disease progression group of arthritis (non RA→RA) compared with non RA patients (non RA→non RA). Third, because age was the only factor that was significantly associated with Pg and Pg was detected even in a half of the patients without PD, similar to previous reports (20), the presence of Pg may not only reflect PD status but also represent the age related alteration of subgingival biofilm.

In this study, we did not observe the positive correlation between Pg and ACPA status. The result is not surprising assuming the small sample size of our study population. In addition, the correlation between Pg and ACPA status may vary depending upon the methods to evaluate Pg infection [14] [15] [34] [35]. In our study, we directly examined bacterial DNA by a PCR based method to evaluate the current bacterial burden for the subsequent development of RA [14] [15] [21], while some studies analyzed anti-Pg antibodies[34] [35]. Furthermore, the correlation between Pg and ACPA or RA may be influenced by the genetic background of the patients such as HLA-DRB1 shared-epitope [34] [36]. The difference in the genetic backgrounds of our Japanese patients and other ethnic groups may partly explain the different results [37] [38].

Our study has several limitations. First, due to our strict inclusion/exclusion criteria, our study population was small and selection bias may exist. Because patients with high arthritis activity and positive ACPA were easily diagnosed as RA and initiated with anti-rheumatic drugs before the referral to our department, those patients were not included in our study. Therefore, our study patients were enriched with ACPA negative (ACPA positivity 44.9%) and relatively mild arthritis patients (average SDAI = 12.5). In addition, we did not find any association between presence of PD defined by the classification of EWP and arthritis severity or introduction of MTX treatment, possibly due to the small sample size. Future studies should be performed in larger scale and in community-hospital based setup to fully determine the role of PD or Pg for RA development. Second, because we defined MTX treatment introduction as the final endpoint, we cannot exclude the possibility that MTX treated patients might include other arthritis patients than RA, although the patients were evaluated by professional rheumatologists [24]. However, as described in the methods section, it was not warranted in our department to wait for the patients to develop into established RA fulfilling the 2010 ACR/EULAR criteria, despite the fact that early MTX treatment can prevent their progression to RA [26].

Despite these limitations, this study is unique in that we conducted a cohort study of treatment naïve arthralgia patients through the parallel evaluations by professional dentists and professional rheumatologists to minimize the error introduced by self-reported PD or antibody responses [2] [3] [35]. To assess the subgingival biofilm independent of the periodontal status, we obtained 8 plaque samples from predetermined tooth sites in each patient[12] and the presence of Pg was analyzed by a highly specific PCR method[21]. We have shown that there is discordance between clinical PD and presence of Pg, and RA development is more associated with clinical state of PD than the presence of Pg.

In conclusion, we have shown that PD, but not the presence of Pg in subgingival biofilm, is significantly associated with arthritis activity and the future requirement of MTX treatment on the diagnosis of RA in treatment naïve arthralgia patients. It will help in designing a strategy to prevent RA development by controlling PD.

## References

[1] Detert J, Pischon N, Burmester GR, Buttgereit F. The association between rheumatoid arthritis and periodontal disease. Arthritis Res Ther. 2010;12(5):218 10.1186/ar3106 21062513 PMC2990988

[2] Demmer RT, Molitor JA, Jacobs DR, Michalowicz BS. Periodontal disease, tooth loss and incident rheumatoid arthritis: results from the First National Health and Nutrition Examination Survey and its epidemiological follow-up study. J Clin Periodontol. 2011;38(11):998–1006. 10.1111/j.1600-051X.2011.01776.x 22092471 PMC3403745

[3] De Pablo P, Dietrich T, McAlindon TE. Association of periodontal disease and tooth loss with rheumatoid arthritis in the US population. J Rheumatol. 2008;35(1):70–6. 18050377

[4] Lundberg K, Wegner N, Yucel-Lindberg T, Venables PJ. Periodontitis in RA-the citrullinated enolase connection. Nat Rev Rheumatol. 2010;6(12):727–30. 10.1038/nrrheum.2010.139 20820197

[5] Wegner N, Lundberg K, Kinloch A, Fisher B, Malmström V, Feldmann M, et al Autoimmunity to specific citrullinated proteins gives the first clues to the etiology of rheumatoid arthritis. Immunol Rev. 2010;233(1):34–54. 10.1111/j.0105-2896.2009.00850.x 20192991

[6] McGraw WT, Potempa J, Farley D, Travis J. Purification, characterization, and sequence analysis of a potential virulence factor from Porphyromonas gingivalis, peptidylarginine deiminase. Infect Immun. 1999;67(7):3248–56. 10377098 10.1128/iai.67.7.3248-3256.1999 PMC116503

[7] Wegner N, Wait R, Sroka A, Eick S, Nguyen K-A, Lundberg K, et al Peptidylarginine deiminase from Porphyromonas gingivalis citrullinates human fibrinogen and α-enolase: implications for autoimmunity in rheumatoid arthritis. Arthritis Rheum. 2010;62(9):2662–72. 10.1002/art.27552 20506214 PMC2941529

[8] Rosenstein ED, Greenwald RA, Kushner LJ, Weissmann G. Hypothesis: The humoral immune response to oral bacteria provides a stimulus for the development of rheumatoid arthritis. Inflammation. 2004. p. 311–8. 10.1007/s10753-004-6641-z 16245073

[9] Scher JU, Abramson SB. The microbiome and rheumatoid arthritis. Nat Rev Rheumatol. 2011;7(10):569–78. 10.1038/nrrheum.2011.121 21862983 PMC3275101

[10] Wu H-J, Ivanov II, Darce J, Hattori K, Shima T, Umesaki Y, et al Gut-residing segmented filamentous bacteria drive autoimmune arthritis via T helper 17 cells. Immunity. 2010;32(6):815–27. 10.1016/j.immuni.2010.06.001 20620945 PMC2904693

[11] Atarashi K, Tanoue T, Oshima K, Suda W, Nagano Y, Nishikawa H, et al Treg induction by a rationally selected mixture of Clostridia strains from the human microbiota. Nature. 2013;500(7461):232–6. 10.1038/nature12331 23842501

[12] Desvarieux M, Demmer RT, Rundek T, Boden-Albala B, Jacobs DR, Sacco RL, et al Periodontal microbiota and carotid intima-media thickness: the Oral Infections and Vascular Disease Epidemiology Study (INVEST). Circulation. 2005;111(5):576–82. 15699278 10.1161/01.CIR.0000154582.37101.15 PMC2812915

[13] Spahr A, Klein E, Khuseyinova N, Boeckh C, Muche R, Kunze M, et al Periodontal infections and coronary heart disease: role of periodontal bacteria and importance of total pathogen burden in the Coronary Event and Periodontal Disease (CORODONT) study. Arch Intern Med. 2006;166(5):554–9. 16534043 10.1001/archinte.166.5.554

[14] Scher JU, Ubeda C, Equinda M, Khanin R, Buischi Y, Viale A, et al Periodontal disease and the oral microbiota in new-onset rheumatoid arthritis. Arthritis Rheum. 2012;64(10):3083–94. 10.1002/art.34539 22576262 PMC3428472

[15] Wolff B, Berger T, Frese C, Max R, Blank N, Lorenz H-M, et al Oral status in patients with early rheumatoid arthritis: a prospective, case-control study. Rheumatology (Oxford). 2014;53(3):526–31. 10.1093/rheumatology/ket362 24273047

[16] Xiong X, Elkind-Hirsch KE, Xie Y, Delarosa R, Maney P, Pridjian G, et al Periodontal disease as a potential risk factor for the development of diabetes in women with a prior history of gestational diabetes mellitus. J Public Health Dent. 2013;73(1):41–9. 10.1111/jphd.12004 23215856

[17] O’Leary TJ, Drake RB, Naylor JE. The plaque control record. J Periodontol. 1972;43(1):38 4500182 10.1902/jop.1972.43.1.38

[18] Tonetti MS, Claffey N. Advances in the progression of periodontitis and proposal of definitions of a periodontitis case and disease progression for use in risk factor research. Group C consensus report of the 5th European Workshop in Periodontology. J Clin Periodontol. 2005;32 Suppl 6:210–3. 16128839 10.1111/j.1600-051X.2005.00822.x

[19] De Oliveira C, Watt R, Hamer M. Toothbrushing, inflammation, and risk of cardiovascular disease: results from Scottish Health Survey. BMJ. 2010 Jan;340:c2451 10.1136/bmj.c2451 20508025 PMC2877809

[20] Wara-aswapati N, Pitiphat W, Chanchaimongkon L, Taweechaisupapong S, Boch JA, Ishikawa I. Red bacterial complex is associated with the severity of chronic periodontitis in a Thai population. Oral Dis. 2009;15(5):354–9. 10.1111/j.1601-0825.2009.01562.x 19371397

[21] Suzuki N, Yoshida A, Nakano Y. Quantitative analysis of multi-species oral biofilms by TaqMan Real-Time PCR. Clin Med Res. 2005;3(3):176–85. 16160072 10.3121/cmr.3.3.176 PMC1237159

[22] Terao C, Hashimoto M, Yamamoto K, Murakami K, Ohmura K, Nakashima R, et al Three Groups in the 28 Joints for Rheumatoid Arthritis Synovitis—Analysis Using More than 17,000 Assessments in the KURAMA Database. PLoS One. 2013;8(3):e59341 10.1371/journal.pone.0059341 23555018 PMC3595245

[23] Aletaha D, Neogi T, Silman AJ, Funovits J, Felson DT, Bingham CO, et al 2010 Rheumatoid arthritis classification criteria: an American College of Rheumatology/European League Against Rheumatism collaborative initiative. Arthritis Rheum. 2010;62(9):2569–81. 10.1002/art.27584 20872595

[24] Kennish L, Labitigan M, Budoff S, Filopoulos MT, McCracken WA, Swearingen CJ, et al Utility of the new rheumatoid arthritis 2010 ACR/EULAR classification criteria in routine clinical care. BMJ Open. 2012;2(5). 10.1136/bmjopen-2012-001117 PMC3488748 23035013

[25] Smolen JS, Landewé R, Breedveld FC, Dougados M, Emery P, Gaujoux-Viala C, et al EULAR recommendations for the management of rheumatoid arthritis with synthetic and biological disease-modifying antirheumatic drugs. Ann Rheum Dis. 2010;69(6):964–75. 10.1136/ard.2009.126532 20444750 PMC2935329

[26] Van Dongen H, van Aken J, Lard LR, Visser K, Ronday HK, Hulsmans HMJ, et al Efficacy of methotrexate treatment in patients with probable rheumatoid arthritis: a double-blind, randomized, placebo-controlled trial. Arthritis Rheum. 2007 May;56(5):1424–32. 17469099 10.1002/art.22525

[27] Machado P, Castrejon I, Katchamart W, Koevoets R, Kuriya B, Schoels M, et al Multinational evidence-based recommendations on how to investigate and follow-up undifferentiated peripheral inflammatory arthritis: integrating systematic literature research and expert opinion of a broad international panel of rheumatologists in the 3E. Ann Rheum Dis. 2011;70(1):15–24. 10.1136/ard.2010.130625 20724311 PMC3002765

[28] Potikuri D, Dannana KC, Kanchinadam S, Agrawal S, Kancharla A, Rajasekhar L, et al. Periodontal disease is significantly higher in non-smoking treatment-naive rheumatoid arthritis patients: results from a case-control study. [Internet]. Annals of the rheumatic diseases. 2012. p. 1541–4. 10.1136/annrheumdis-2011-200380 22875903

[29] Martinez-Martinez RE, Abud-Mendoza C, Patiño-Marin N, Rizo-Rodríguez JC, Little JW, Loyola-Rodríguez JP. Detection of periodontal bacterial DNA in serum and synovial fluid in refractory rheumatoid arthritis patients. J Clin Periodontol. 2009;36(12):1004–10. 10.1111/j.1600-051X.2009.01496.x 19929953

[30] Arend WP, Firestein GS. Pre-rheumatoid arthritis: predisposition and transition to clinical synovitis. Nat Rev Rheumatol; 2012 Oct;8(10):573–86. 10.1038/nrrheum.2012.134 22907289

[31] Hashimoto M, Hirota K, Yoshitomi H, Maeda S, Teradaira S, Akizuki S, et al Complement drives Th17 cell differentiation and triggers autoimmune arthritis. J Exp Med. 2010;207(6):1135–43. 10.1084/jem.20092301 20457757 PMC2882841

[32] Darveau RP. Periodontitis: a polymicrobial disruption of host homeostasis. Nat Rev Microbiol. 2010;8(7):481–90. 10.1038/nrmicro2337 20514045

[33] Ozmeriç N, Preus NR, Olsen I. Genetic diversity of Porphyromonas gingivalis and its possible importance to pathogenicity. Acta Odontol Scand. 2000;58(4):183–7. 11045373 10.1080/000163500429190

[34] Mikuls TR, Thiele GM, Deane KD, Payne JB, O’Dell JR, Yu F, et al Porphyromonas gingivalis and disease-related autoantibodies in individuals at increased risk of rheumatoid arthritis. Arthritis Rheum. 2012;64(11):3522–30. 10.1002/art.34595 22736291 PMC3467347

[35] Hitchon CA, Chandad F, Ferucci ED, Willemze A, Ioan-Facsinay A, van der Woude D, et al Antibodies to porphyromonas gingivalis are associated with anticitrullinated protein antibodies in patients with rheumatoid arthritis and their relatives. J Rheumatol. 2010;37(6):1105–12. 10.3899/jrheum.091323 20436074

[36] Lundström E, Källberg H, Alfredsson L, Klareskog L, Padyukov L. Gene-environment interaction between the DRB1 shared epitope and smoking in the risk of anti-citrullinated protein antibody-positive rheumatoid arthritis: all alleles are important. Arthritis Rheum. 2009;60(6):1597–603. 10.1002/art.24572 19479873 PMC2732897

[37] Terao C, Ohmura K, Kochi Y, Ikari K, Maruya E, Katayama M, et al A large-scale association study identified multiple HLA-DRB1 alleles associated with ACPA-negative rheumatoid arthritis in Japanese subjects. Ann Rheum Dis [Internet]. 2011;70(12):2134–9. 10.1136/annrheumdis-2011-200353 21873689

[38] Marotte H, Farge P, Gaudin P, Alexandre C, Mougin B, Miossec P. The association between periodontal disease and joint destruction in rheumatoid arthritis extends the link between the HLA-DR shared epitope and severity of bone destruction. Ann Rheum Dis. 2006;65(7):905–9. 16284099 10.1136/ard.2005.036913 PMC1798215

